# The effectiveness of locally-prepared peritoneal dialysate in the management of children with acute kidney injury in a south-east Nigerian tertiary hospital

**DOI:** 10.4314/ahs.v22i4.74

**Published:** 2022-12

**Authors:** Ngozi R Mbanefo, Samuel N Uwaezuoke, Ugo N Chikani, Ada I Bisi-Onyemaechi, Uzoamaka V Muoneke, Odutola I Odetunde, Henrietta U Okafor

**Affiliations:** Department of Paediatrics, University of Nigeria Teaching Hospital Ituku-Ozalla, Enugu, Nigeria

**Keywords:** Peritoneal dialysis, renal replacement therapy, acute kidney injury, children, dialysate, developing country

## Abstract

**Background:**

Peritoneal dialysis (PD) is the preferred mode of renal replacement therapy (RRT) in children with acute kidney injury (AKI). The gold standard remains the use of commercially-prepared PD fluid. In resource-poor nations, its availability and affordability remain a challenge.

**Aim:**

This study aims to report the effectiveness of locally-prepared PD fluid in the management of AKI in a south-east Nigerian tertiary hospital.

**Subjects and Methods:**

This was a retrospective study conducted at the paediatric ward of the University of Nigeria Teaching hospital, Enugu. The case records of 36 children seen over three years, diagnosed with AKI and requiring PD were reviewed. The retrieved information comprised biodata, aetiology of AKI, indications for PD, pre-and post-dialysis estimated glomerular filtration rate (eGFR) and patient outcomes.

**Results:**

The children (20 males and 16 females) were aged 3 to 36 months with a mean age of 9.92 ± 6.29 months. The common aetiologies of AKI were septicemia (30.6%), hemolytic uremic syndrome (19.4%), and toxic nephropathy (16.7%). The frequent indications for PD were uremic encephalopathy (58.3%) and severe metabolic acidosis (38.8%). The pre-and post-dialysis mean urine flow rate was 0.16 + 0.13 and 2.77 + 0.56 ml/kg/hour respectively. The eGFR before PD, at discontinuation, and a week later was 6.06 + 2.87, 24.44 + 15.71 and 59.07 + 22.22 mls/min/1.73m2 respectively.

**Conclusion:**

PD with locally-prepared dialysate is safe, effective and a life-saving alternative in the management of AKI in children

## Introduction

In critically-ill children, occurrence of acute kidney injury (AKI) is consistent with poor outcomes including possible long-term sequelae such as hypertension and chronic kidney disease (CKD).^1^, ^2^ Early detection of and intervention for AKI are thus important in improving prognosis. Where conservative management fails, renal replacement therapy (RRT) is promptly required to significantly reduce morbidity and mortality.

Peritoneal dialysis (PD) is the preferred mode of RRT in children with AKI,^3^ and the gold standard remains the use of commercially-prepared dialysis fluid.^4^ However, the availability and affordability of the commercially-prepared dialysate in resource-poor countries remains a challenge. This poor access to the conventional dialysate contributes significantly to AKI-associated mortality. Thus, the effectiveness of a locally-prepared dialysate is pivotal for stemming this trend in developing countries within the African setting.

In Nigeria, the cost of commercially-prepared dialysate is quite exorbitant considering the absence of production plants in the country, cost of importation and storage facilities, and if available, limitation of accessibility in low-income areas. More than 82.9 million Nigerians (about 40% of the total population) live on less than one dollar per day.^5^ Therefore, with the poor health insurance scheme and the high out-of-pocket expenditures, reduction in the overall cost of health-care needs and services reduces despondency and improves consent.

Findings from other countries have shown that the locally prepared PD fluid is as effective as the commercially-prepared dialysate,^6^–^8^ and therefore should be considered as a life-saving alternative. In Nigeria, authors from the south-west 9, 10 and south-south regions 11 have reported the effectiveness with its use. The aim of our study is to document the effectiveness and safety of such improvised dialysate in our setting (the south-eastern part of Nigeria). The demonstration of good outcomes will justify its routine use towards reducing AKI-related mortality. Importantly, it will encourage the alternative use of locally-prepared dialysate where the commercially-prepared type is not available.

## Subjects and Methods

This was a retrospective observational descriptive study conducted at the paediatric ward of the University of Nigeria Teaching hospital (UNTH), Ituku-ozalla, Enugu. The UNTH is a tertiary hospital and a referral centre established mainly for the people living in the south-eastern part of Nigeria and beyond.

The case records of thirty-six children seen over 3 years (from 2017 to 2019), diagnosed with AKI and requiring PD were reviewed. AKI was defined as the abrupt or sudden (occurring within hours to days) deterioration or impairment in renal function manifesting as accumulation of nitrogenous waste products of metabolism (increase in blood urea nitrogen and creatinine level), fall in glomerular filtration rate, reduction in urine output, fluid retention and electrolyte imbalance. Diagnosis and classification of the affected children was done using the RIFLE/KDIGO criteria^12^,^13^ and all the patients who had the procedure were in stages F and 3 respectively. Those with incomplete records were excluded. The data extracted from the case folders included age, gender, primary cause of AKI, duration of symptoms of AKI before presentation, renal function tests before and after the procedure, indications for the PD, type of dialysate and details of the reconstitution, duration of dialysis and number of sessions, dialysis-related complications and outcome. All the patients were followed up from the point of admission to at least 12 weeks after discharge. Data obtained was anonymized hence an expedited ethical clearance was obtained from the Health Research & Ethics Committee (HREC) of the hospital. (Approval number: UNTH/HREC/2021/07/234).

### Dialysate preparation

Routinely, the PD fluid was freshly prepared by the bedside in a sterile bag under strict sterile condition. The fluid was composed of 440 mls of 5% D/W, 60 mls of intravenous 8.4% sodium bicarbonate, and 1000 mls of normal saline to make up 1500 mls of the dialysate and comprising 1.5% dextrose strength solution. To each 1.5 L of the reconstituted fluid, 250 IU – 750 IU of heparin was added; a lower dose was used in cases of thrombocytopenia and/or increased international normalized ratio (INR). A hypertonic dextrose solution of 2.5% was prepared by substituting 10% D/W for 5% D/W for those with anasarca. Addition of 20mls of 50% dextrose to each litter of 1.5% or 2.5% increases the strength by 0.5%. Intravenous ceftazidime (Fortum) at 125mg/l (187.5mg in 1500mls) and vancomycin at 20mg/l (30mg in 1500mls) were added as prophylaxis against the development of peritonitis.

### Routine PD Procedure

At the bed side, the Kimal or rigid catheter was inserted 2 cm below the umbilicus using the Seldinger technique^14^ after a local disinfection of the site. The continuous spilling of a warm and clear peritoneal fluid signified a successful insertion. Any resistance with filling and/or interruption with flow indicated improper catheter placement or dislodgement. An inspection of the site for possible leakage and the colour of the fluid to ensure no intra-peritoneal bleeding or fecal contamination were done. A bio-patch was applied immediately at the point of entry of the catheter before covering with sterile gauze in order to limit colonization by bacteria. The prepared fluid was delivered to a child through a sterile buretrol to ensure that the correct measured volume was administered. With the aid of a 3-way tap, the process of filling, dwelling and emptying was manually regulated. In all of these connections, a closed system is created and maintained with strict asepsis. For the sessions, 20mls/kg was used to determine the fill volume. In cases of severe respiratory distress, a smaller volume of 10mls /kg was applied and increased when tolerability improved to up to 30mls/kg. The dialysate was usually allowed to dwell for 45 to 60 minutes and drained over 30 minutes. Due to the unavailability of the PD automated machine, the exchanges were carried out manually by the resident doctors (not more than 2) on duty. The number of sessions, the dwell time, volume and colour of the effluent, and fluid balance were noted and recorded in a PD chart prepared for each individual. All the patients received an intravenous cephalosporin or carbapenem with doses adjusted according to the estimated glomerular filtration rate (eGFR) as prophylaxis with the first dose given 30minutes to one hour before the procedure and continued until the end of the dialysis (3–5days). Also, intravenous 10% calcium gluconate is given at a dose of 0.5–1ml/kg but not exceeding 10mls daily for 48–72 hours. Monitoring included measurement of the vital signs, random blood glucose level and the urine flow rate. Within 24 hours of onset of dialysis, a repeat serum electrolyte, urea & creatinine (SEUCr) was estimated and monitored serially thereafter. Dialysis was discontinued once there was a significant and/or sustained improvement with the urine flow rate to at least 1.5 ml to 2 mls/kg/hour, fall in the blood urea nitrogen (BUN) and serum creatinine levels, resolution of electrolyte derangements, and a general improvement in the patient's wellbeing.

### Data analysis

Data were analysed on descriptive statistics using SPSS version 23 for windows. Descriptive statistics which include frequency and percentages were used to summarize categorical variables (qualitative data) while means and standard deviations were obtained for continuous variables (quantitative data). Means of continuous variables were compared using t test and ANOVA. P-value < 0.05 was considered statistically significant. Results were presented in tables.

## Results

### Patients' demographics

During the period under review, a total of thirty-six children who were diagnosed with AKI had PD with the bed-side locally-prepared PD fluid; of which twenty were males (55.6%) and sixteen (44.4%) were females. The children were aged 3 to 36 months with a mean age of 9.92 ± 6.29 months. The majority of the children (80.6%) were aged less than one year as shown in Table 1.

**Table 1:** The sociodemographic characteristics, aetiology of acute kidney injury, and indications for peritoneal dialysis

Variables	Frequency (n)	Percentage (%)
Age in months* • < 6 • 7–12 • > 12	11 18 7	30.6 50.0 19.4
**Gender** • Male • Female	20 16	55.6 44.4
**Aetiology of AKI** • Septicaemia	11	30.6
• Haemolytic uremic syndrome	7	19.4
• Toxic nephropathy	6	16.7
• Algid malaria	5	13.9
• Pigment nephropathy	4	11.2
• Post-cardiac surgery	3	8.3
**Indications for PD** • Oliguria/anuria	36	100
• Azotemia	36	100
• Severe metabolic acidosis	21	58.3
• Hyperkalemia	17	47.2
• Uremic encephalopathy	14	38.8
• Pulmonary oedema	10	27.7

* Mean age ± SD = 9.92 ± 6.29 (minimum age = 3 months, maximum age = 36 months) AKI, acute kidney injury PD, peritoneal dialysis

### Aetiology of AKI

While septicaemia seen in 30.6% of affected children was found to be the commonest aetiological factor for AKI, only about 8.3% of those who had surgery (post cardiac) had AKI. Other important causes of AKI noted to be mainly intrinsic in origin included hemolytic uremic syndrome (19.4%), toxic nephropathy (16.7%) and pigment nephropathy (11.2%). Malaria was implicated in about five (13.9%) patients (Table 1).

### Dialysis Indications

As shown in Table 1, the indications for instituting dialysis were multifactorial. All the thirty-six children (100%) were either oliguric or anuric, and had varying degrees of generalized body swelling, elevated BUN and serum creatinine levels at presentation. In addition to these factors, clinical assessment showed that twenty-one (58.3%) of the patients had severe and symptomatic metabolic acidosis, seventeen (47.2%) had moderate-to-severe hyperkalemia while fourteen (38.8%) patients had uremic encephalopathy. Only ten (27.7%) of the affected children had pulmonary oedema. The mean (SD) number of sessions of dialysis was 28.1 (13.9) while the mean (SD) duration was 2.8 (0.9) days. The mean (SD) number of exchanges per day was 9.7 (3.2). The mean (SD) duration of symptoms of AKI before presentation was 3.1 (1.8) days and the range was 1 – 11.

### Effectiveness of the PD fluid

After 48 hours of dialysis, a repeat renal function test showed a significant and gradual restoration of the serum electrolytes, BUN and creatinine towards normal values. The mean eGFR improved from 6.06 ± 2.87 at presentation to 24.44 ± 15.71 on suspension of dialysis and further to 59.07 ± 22.22 one-week post dialysis. Also, there was a sustained increment in the mean urine flow rate from 0.16 ± 0.13 ml/kg/hour at presentation to 2.77 ± 0.56 ml/kg/hour on suspension of the procedure (t = 26.099, p < 0.001), (Tables 2 and 3). Table 2 shows a significant increase in sodium, bicarbonate, chloride, eGFR and urine flow rate after the use of the locally-prepared PD fluid in children with AKI (p < 0.05), while a significant decrease was found in potassium, BUN and creatinine levels. The serum creatinine level reduced significantly after the use of the locally-prepared dialysate for PD; and further at 1st week of follow-up (F = 194.389, p < 0.001). In Table 3, serum creatinine reduced significantly after the use of the locally-prepared PD fluid; and reduced further at 1st week of follow-up (F = 194.389, p < 0.001).

**Table 2:** Comparison of electrolytes, blood urea nitrogen, serum creatinine, estimated glomerular filtration rate and urine flow rate before and after use of locally-prepared dialysate

	Before peritoneal dialysis Mean ± SD	After peritoneal dialysis Mean ± SD	T	p-value
Sodium (mmol/l)	127.79 ± 7.57	138.10 ± 2.38	8.309	< 0.001
Potassium (mmol/l)	4.86 ± 1.29	3.69 ± 0.46	4.616	< 0.001
Bicarbonate (mmol/l)	12.53 ± 2.44	18.81 ± 1.85	19.409	< 0.001
Chloride (mmol/l)	95.01 ± 9.63	101.09 ± 3.16	3.624	0.001
BUN (mmol/l)	34.21 ± 6.33	13.78 ± 4.02	20.287	< 0.001
Creatinine (mg/dl)	5.57 ± 1.65	2.03 ± 0.67	14.104	< 0.001
eGFR(mls/min/1.73m^2^)	6.06 ± 2.87	24.44 ± 15.71	7.496	< 0.001
UFR (ml/kg/hr)	0.16 ± 0.13	2.77 ± 0.56	26.099	< 0.001

SD, standard deviation BUN, blood urea nitrogen eGFR, estimated glomerular filtration rate UFR, urine flow rate

**Table 3:** Comparison of serum creatinine and estimated glomerular filtration rate before and after the use of locally-prepared dialysate

	Before PD Mean ± SD	After PD Mean ± SD	1st week after Mean ± SD	F	p-value
Creatinine (mg/dl)	5.57 ± 1.65	2.03 ± 0.67	0.82 ± 0.22	194.389	< 0.001
eGFR mls/min/1.73m^2^	6.06 ± 2.87	24.44 ± 15.71	59.07 ± 22.22	103.144	< 0.001

SD, standard deviation eGFR, estimated glomerular filtration rate PD, peritoneal dialysis

### Procedure-related complications and outcomes

A significant number (20; 58.3%) of patients had no complications. Specifically, there was no case of peritonitis. Only nine (25%) of the children had catheter-related complications from leakages and blockage. It is important to mention that two (5.6%) children had hyperglycemia. A very high survival rate of 94.4% and low mortality rate of 5.5% were seen. All the children were followed up in the clinic from one week after discharge to several months later.

## Discussion

AKI defined as the sudden deterioration in renal function and characterized by a decline in GFR, retention of urea and other nitrogenous waste products, and dysregulation of fluid and electrolytes, could be devastating once established in any critically ill child. Since there are no reported specific medications for the management of AKI, RRT remains the only treatment option to limit avoidable deaths.^15^–^17^ Due to several factors including but not limited to better hemodynamic tolerability, ease of catheter insertion, minimal technological ability, better preservation of residual kidney function, PD remains the preferred mode of RRT in children.^18^–^21^ As a developing nation with economic challenges, it is pertinent to determine the effectiveness of locally-prepared dialysate in improving clinical outcomes if AKI occurs.

Findings from the present study showed that many of the PD procedures were done during the first 3 months of the year when acute gastroenteritis was rife^22^–^24^, a major cause of pre-renal AKI. During this dry period, there is usually lack of access to clean and safe water causing unhygienic practices, poor sanitation, contamination of food and drinking water. Secondly, the dusty weather encountered around same time encouraged the spread of rotaviruses.^23^ These risk factors^24^ cause an upsurge in diarrhoeal diseases and subsequent development of volume depletion which is complicated by AKI if prompt fluid replacement is not instituted. Unlike reports from Esezobor et al.,^9^ and Ademola et al,^10^ the majority of the children who accessed the procedure were males (55.6%) and infants (80.6%). In agreement with Esezobor et al,^9^ the aetiology of AKI was predominantly due to sepsis and intrinsic renal diseases such as hemolytic uremic syndrome, toxic nephropathy, and pigment nephropathy. While the septicaemia commonly followed an episode of an acute gastroenteritis, toxic nephropathy occurred as a result of an intervention with indiscriminate use of medications especially multiple nephrotoxic antibiotic therapy and herbal concoctions. Pigment nephropathy was seen in older (more than 6 months) male children who were also found to be Glucose 6 Phosphate Dehydrogenase deficient and in addition to other clinical features also had jaundice and hematuria.

The indication for PD in most patients was multifactorial as published earlier from our center^25^. In addition to oligo-anuria and azotemia seen in all of the affected children, the development of uremic encephalopathy, pulmonary oedema from several intravenous fluid therapies in an attempt to force diuresis and fluid retention, seizures from multiple electrolyte derangements, and symptomatic metabolic acidosis typically necessitated referral and immediate intervention. Most of the affected children were seen within 48 hours to 72 hours of developing these symptoms. The only patient who presented very late (more than a week) was due to delay in referral. Our findings confirmed that AKI in children can be effectively managed with a favourable outcome with the use of locally-prepared dialysate. It is worthy to mention that in addition to PD, other comorbidities present were concurrently treated. Blood transfusion was given in aliquots of 5 – 7.5mls/kg alternate daily to those with severe anaemia. Seizures were aborted and controlled with intravenous midazolam (at 0.1mg/kg not exceeding 5mg) and phenobarbitone at (5mg/kg/day in 2 divided doses). Titrated intravenous aminophylline at 5mg/kg/day was given for renal reperfusion and in pulmonary oedema. Usually, intranasal oxygen was given in cases of poor saturation. Systemic antibiotics were administered to those with septicaemia for up to 7 days. With some clinical improvement on day 2 of PD, nasogastric tube was passed for feeding and administration of probiotic and zinc for children with diarrhoeal episodes and amlodipine for control of hypertension. Fluid therapy is restricted to previous day's loss plus insensible loss while intravenous frusemide was given at 2–3mg/kg/day in 4 divided doses. On clinical evaluation, the resolution of respiratory distress and withdrawal of possible oxygen therapy, regaining of consciousness, absence of seizure, resolution of body swelling and improvement in urine output indicated an effective response. The laboratory finding of a significant and steady fall in serial serum BUN and creatinine level and a corresponding rise in the eGFR supported its effectiveness.

Unlike the reports from other centres in Nigeria, 9–11 no case of peritonitis was reported in this study. This finding was attributed to many factors which included strict aseptic procedure (during catheter insertion and fluid reconstitution), use of antibiotics either systemic and/or as an additive (cephalosporin or carbapenem, and vancomycin), application of bio patch at the point of entry, immediate discontinuation and removal of the catheter once positive response is achieved and sustained (usually not more than three days), and minimal handling (as only two resident doctors manually coordinate the process).

A very high survival rate of 94.4% seen in our centre when compared to others^9^–^11^ was due to prompt intervention enabled by provision of catheters and other necessary consumables and medications required for fluid reconstitution within the pharmacy outlet on the ward. Secondly, a positive hospital policy of no demand for immediate payment from caregivers of such category of patients allowed for quick initiation of the procedure. Thirdly, the PD catheter insertion by the nephrologists hastens the process and avoids further delay usually encountered if the surgeons were to be invited for the procedure. The low mortality rate of 5.5% seen in two patients occurred within six hours of the procedure where they both had less than five sessions of dialysis. The deaths were attributed to delay in presentation and possible presence of other morbidities.

## Conclusions

The locally-prepared PD fluid when applied with the right expertise and technique is safe and as effective as the commercially-prepared dialysate. In resource-poor settings, we advocate its use as an alternative resource for RRT. Individual paediatric nephrology centres in developing countries are encouraged to domesticate the use of these non-commercially-prepared PD fluids in the management of AKI. This will increase accessibility to RRT among the low-income clientele in these settings.

## Study limitations

Few numbers of patients were reviewed during the study. Secondly, the retrospective nature of the study led to the likelihood of missing data. Thirdly, it is a single centre study.
